# Construction of Reverse Genetics System for Feline Calicivirus FCV‐BJ616 and Proteomic Analysis

**DOI:** 10.1002/mbo3.70226

**Published:** 2026-01-28

**Authors:** Chunmei Xu, Jingjie Zhao, Hao Liu, Haotian Gu, Xinming Tang, Lin Liang, Jiabo Ding, Shaohua Hou, Xiaomin Zhao, Ruiying Liang

**Affiliations:** ^1^ Key Laboratory of Animal Biosafety Risk Prevention and Control (North) & Key Laboratory of Veterinary Biological Products and Chemical Drugs of MARA, Institute of Animal Science, Chinese Academy of Agricultural Sciences Beijing China; ^2^ College of Veterinary Medicine Northwest A&F University Yangling Shaanxi China

**Keywords:** feline calicivirus, proteomics, reverse genetics of viruses

## Abstract

Feline calicivirus (FCV) is a primary cause of upper respiratory tract infections and oral ulcerative disease in cats and exhibits substantial genetic diversity that complicates prevention and control. In this study, we isolated the FCV‐BJ616 strain, established a reverse‐genetics system, and investigated its pathogenic mechanisms, thereby providing a foundation for antibody‐based therapies and broad‐spectrum vaccine development. The virus was purified by three rounds of plaque cloning, and its morphology was examined by electron microscopy. VP1 expression was confirmed by immunofluorescence and Western blotting. Using integrated systems‐biology and reverse‐genetics approaches, an infectious clone of rFCV‐BJ616 was successfully assembled and rescued, exhibiting genetic stability comparable to that of the parental strain. In vivo infection experiments showed that rFCV‐BJ616 retained wild‐type virulence, causing persistent high fever, weight loss, and multiorgan pathology in infected cats. Proteomic analysis indicated that infection with FCV‐BJ616 or rFCV‐BJ616 markedly activated cytokine‐mediated inflammatory signaling pathways. Both FCV‐BJ616 and rFCV‐BJ616 significantly upregulated the expression of IL‐8, S100A8/A9, and TLR3, which are associated with acute inflammation and tissue damage. Furthermore, elevated IFN‐β levels concomitant with STAT1 downregulation suggested a transient attenuation of antiviral signaling during early immune activation. These findings were corroborated by ELISA‐based validation of serum cytokine profiles. Collectively, this study provides new insights into the molecular pathogenesis and evolution of FCV‐BJ616 and establishes a robust reverse‐genetics platform for precise genome manipulation and future vaccine development.

## Graphical abstract

This study successfully isolated the FCV‐BJ616 strain derived from cats and established a reverse genetics system. In vivo experiments showed that rFCV‐BJ616 exhibits wild‐type virulence, leading to high fever, weight loss, and multi‐organ lesions in infected cats. Proteomic analysis indicated that both rFCV‐BJ616 and FCV‐BJ616 share similar regulatory pathways. Overall, this research provides new insights into the molecular pathogenic mechanisms and evolutionary history of FCV‐BJ616, while establishing a reliable reverse genetics platform to facilitate future vaccine development.

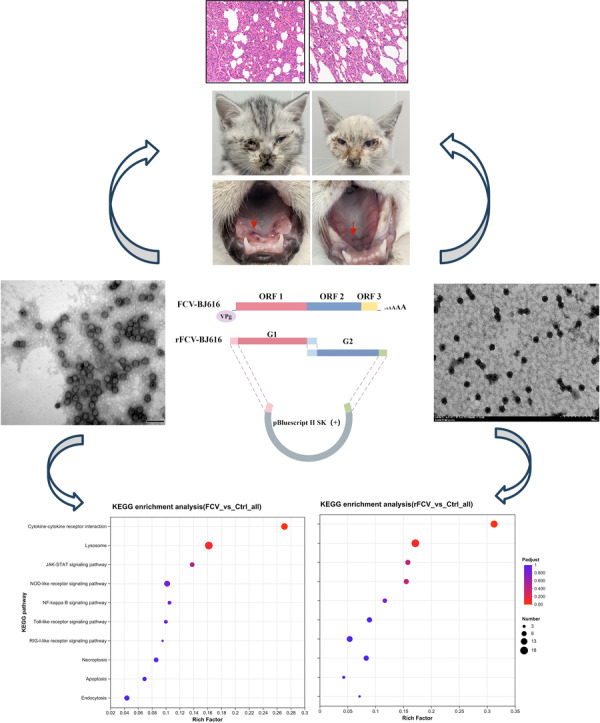

## 1 Introduction

The Caliciviridae family consists of single‐stranded, positive‐sense RNA viruses, with particles ranging from 27 to 40 nm in diameter and genomes between 7.4 and 8.3 kilobases, all enclosed in icosahedral capsids (Binn et al. 2018; Matsvay et al. 2022; Mikalsen et al. 2014; Vinjé et al. 2019). These viruses infect a wide range of hosts, including mammals, fish and birds, and pose significant health risks. Although members such as human norovirus (HuNoV) are major causes of acute gastroenteritis(Raymond et al. 2023), most caliciviruses are difficult to culture in vitro. Only a few, including feline calicivirus (FCV) and Murine norovirus (MNV) can be efficiently cultured(Chen et al. 2025; Kolawole et al. 2016), making FCV an important model for studying calicivirus replication and pathogenesis.

FCV is the primary pathogen responsible for feline upper respiratory infections and oral ulcers. In recent years, outbreaks of feline calicivirus–associated virulent systemic disease (FCV‐VSD), a highly virulent clinical syndrome, have been documented in North America, Europe, and Asia, with reported case‐fatality rates of 40%–70%(Bordicchia et al. 2021; Brunet et al. 2019; Caringella et al. 2019; Park et al. 2024; Y. Zhang et al. 2025). Despite the widespread use of commercial vaccines, cross‐protection against VSD strains remains limited due to ongoing mutations in the VP1 capsid protein. The FCV genome encodes a major capsid protein, VP1, whose hypervariable region (HVR) plays a central role in antigenic drift and immune evasion(Abente et al. 2013; Brunet et al. 2019; Yang et al. 2025). Specific amino acid substitutions in VP1 have been linked to virulence (Brunet et al. 2019), yet the underlying molecular mechanisms remain poorly defined.

Reverse genetics is a powerful tool for studying the pathogenicity and mutation mechanisms of RNA viruses. An FCV reverse genetics platform has been successfully developed using various approaches. For instance, capped FCV RNA is transcribed in vitro and transfected into susceptible cells (Sosnovtsev and Green 1995), and the FCV cDNA plasmid driven by the EF1α promoter is also transfected into susceptible cells (Cheng et al. 2023). Additionally, using the circular polymerase extension reaction (CPER) method, researchers successfully constructed a full‐length cDNA molecular clone of the VS‐FCV SH/2014 strain (X. Wang et al. 2024). T7 RNA polymerase is widely used in molecular biology because of its high transcription efficiency and specificity. Transfecting full‐length cDNA into T7 polymerase‐sensitive cells constitutes an effective gene expression strategy. The core of this method lies in the highly efficient transcription system driven by the T7 promoter. Studies have shown that efficient transcription within cells significantly enhances the expression levels of the target gene (Conrad et al. 2020).

We integrated virology, reverse genetics, and multi‐omics to investigate FCV virulence. We isolated a highly virulent strain (FCV‐BJ616), characterized its genomic evolution and putative virulence mutations, and rescued an infectious clone using a T7‐based system to assess genetic stability and pathogenicity in cats. Proteomic and pathological analyses defined host responses and systemic inflammatory pathways underlying FCV‐induced VSD. This work establishes a robust reverse‐genetics platform to dissect viral gene function, clarify virulence and host adaptation, and inform the rational design of attenuated or broad‐spectrum FCV vaccines.

## 2 Material and Method

### 2.1 Plasmids, Cells and Viruses

This study utilized the plasmid pBluescript II SK ( + ), F81 cells, and BSR T7/5 cells (BHK‐21 clone stably expressing T7 RNA polymerase), all of which were maintained in our laboratory. F81 cells (cat kidney cells) were cultured in DMEM medium supplemented with 10% FBS (Gibco) at 37°C in a 5% CO2 atmosphere. The FCV‐BJ616 strain was isolated in 2024 from a clinical sample of a severely ill cat exhibiting systemic symptoms in Beijing.

### 2.2 Viral Isolation and Plaque Purification

F81 cells were seeded in 6‐well plates at a concentration of 7 × 10⁵ cells/mL. Once a monolayer was formed, the culture medium was removed, and the cells were washed with PBS. A 200 μL gradient‐diluted viral suspension was added, followed by a 90‐min adsorption period at 37°C with 5% CO₂. The viral suspension was discarded, and the cells were washed again. Next, 2 mL of pre‐warmed first‐layer agar solution (4% low‐melting‐point agarose and 5% FBS‐2×DMEM in equal volumes) was added. After solidification, the plates were inverted for culturing, with observations made every 12 h. When significant lesions developed, a second layer of agar solution containing 0.01% neutral red was applied. The plates were then returned to an upright position and cultured, with observations conducted every 3 h. Once the lesions had formed, the infected spots were picked. This purification process was repeated three times to obtain the cloned viral strain.

### 2.3 FCV Genome Amplification and Genetic Evolution Analysis

GenBank‐verified FCV sequences were used to analyze differential expression using MEGA7.0. Primers were designed and synthesized by Beijing Qingke. Four primer pairs were employed to amplify the FCV fragment (Table 1). This fragment was recovered via gel electrophoresis and cloned into the pEASY‐Blunt Kit. The recombinant clones were then transformed into Trans1‐T1 cells. assembled using DNASTAR SeqMan after sequencing. Finally, we performed a sequence alignment of the full genome and VP1 sequences of FCV‐BJ616 with 69 FCV sequences uploaded to NCBI between 2020 and 2025 using MEGA7.0.

**Table 1:** FCV identify and whole genome primer sequences.

Primer name	Sequence(5′ → 3′)	bp
FCV‐JD‐F	GAATTGGCTAARATCTTRCATGA	452
FCV‐JD‐R	GGRGTTTCAGAGTTDGARGTCA	
1 F	GTAAAAGAAATTTGAGACAATGT	2030
1 R	TAGTTTGGGCCAAGGAGTCAATATTTAAGAAAG	
2 F	TGACTCCTTGGCCCAAACTATGAAGCAGGA	2017
2 R	AGCCAGGTGATCGATAAGCATCTTCTGGACTCG	
3 F	TGCTTATCGATCACCTGGCTGGCTTTGTACCTATG	2023
3 R	CTGGAGGCACAACAATTGCTGCCAACTTCCCA	
4 F	AGCAATTGTTGTGCCTCCAGGCGTTCA	1688
4 R	CCCTGGGGTTAGGCGCAAGAGCGGCAGC	

### 2.4 Construction of Recombinant cDNA Clones

The full‐length cDNA clone of FCV‐BJ616 was assembled in a pBlueScript II (+) vector. The viral coding sequence, retaining its native 5′ and 3′ untranslated regions (UTRs) to preserve cis‐acting elements essential for replication and translation, was positioned under the control of a T7 RNA polymerase promoter to drive in vitro transcription. To ensure the generation of authentic 3′ viral genomic ends, a hepatitis delta virus ribozyme (HdvRz) sequence was inserted directly downstream of the 3′ UTR, followed by a synthetic poly(A) tail to enhance the stability and translational efficiency of the transcribed RNA. To validate the modified pBlueScript II (+) vector, the eGFP gene was inserted into the 5′ UTR of FCV, and fluorescence expression was monitored. Primers were designed using DNA Man and DNA Star based on the FCV‐BJ616 sequence, and the digestion site was analyzed with NovoPro. A mutation (G → A) was introduced at position 4505 in the *Xho* I site as a genetic marker. The primers were synthesized by Bgi Genomics Co. Ltd. The FCV‐BJ616 genome was divided into g1‐g4 fragments, with G1 and G2 obtained through gel recovery and fusion PCR. These fragments were then seamlessly cloned into the modified pBlueScript II (+) vector. PCR verification and sequencing confirmed the successful construction of the pBlueScript II ( + )‐G1G2 vector.

### 2.5 Rescue of Recombinat FCV

Recombinant pBluescript II SK ( + )‐G1G2 was transfected into single‐layer BSR T7/5 cells using Lipofectamine 3000. The recombinant FCV virus was harvested 48 h post‐transfection.

### 2.6 Viral Titration and In Vitro Growth Curve

To determine the TCID_50_, F81 cells were cultured in 96‐well plates for 24 h. FCV was serially diluted tenfold (10^−1^ to 10^−8^) in DMEM and inoculated. After 1 h of adsorption at 37°C, the dilutions were removed, cells washed three times with DMEM, and fresh DMEM with 1% FBS was added. CPE was monitored after 48 h at 37°C, and TCID_50_ was calculated using the Reed‐Muench method.

For growth curve assessment, F81 cells were cultured in 12‐well plates for 24 h. Cells were infected with FCV at an MOI of 0.1 and incubated at 37°C. After infection, cells were washed three times with DMEM and replaced with DMEM containing 1% FBS. Cell lysates and supernatants were collected at 6, 12, 24, 48, and 72 h post‐infection to determine TCID_50_.

### 2.7 Transmission Electron Microscopy

After culturing the virus to the 6th generation, the virus was collected in 200 mL of solution after three freeze‐thaw cycles. The virus was centrifuged at 4°C at 5000 g for 15 min to remove debris. The supernatant was transferred to pre‐disinfected ultracentrifuge tubes and centrifuged at 4°C at 30,000 g for 1 h. The precipitate was resuspended in 500 μL PBS and overlaid onto a 20%–60% sucrose gradient, followed by centrifugation at 100,000 g for 7 h. Under a dark chamber spotlight, the viral bands were aspirated, adsorbed on a copper grid for 1 min, washed twice with PBS, negatively stained with 1% phosphotungstic acid for 1 min, and observed under an electron microscope.

### 2.8 Western Blotting

Cultivate cells in 6‐well plates as monolayers for 24 h. Infection with FCV was performed at 37°C. After 1‐h adsorption, the supernatant was removed and cells were washed three times with DMEM. Fresh DMEM supplemented with 1% FBS was added. Supernatant or cells were collected post‐infection. Cells were lysed in RIPA buffer (Beyotime) at low temperature, and the lysate was centrifuged at 12,000 rpm for 10 min to remove precipitates. Equal volumes of lysate were subjected to 12% SDS‐PAGE and transferred to 0.2 μm PVDF membranes (Millipore). The 5% skim milk was incubated for 1 h. Subsequently, mouse anti‐VP1 monoclonal antibody (1:2000; Preparation and preservation in this laboratory) and anti‐β‐actin monoclonal antibody (1:2000; Abcam; ab6276) were incubated overnight at 4°C. Following this, Goat Anti‐Mouse IgG H&L (HRP) (1: 10,000; Abcam; ab6789) was incubated for 1 h at room temperature. Results were visualized using the Odyssey CLx system.

### 2.9 Immunofluorescence Assay

F81 cells grown on coverslips were infected with Feline Calicivirus (FCV, MOI = 1) for 24 h. Cells were fixed with 4% paraformaldehyde, permeabilized with 0.1% Triton X‐100, and blocked with 5% BSA. Primary antibody incubation used mouse anti‐VP1 IgG (1:200, Preparation and preservation in this laboratory), followed by Alexa Fluor 488‐conjugated goat anti‐mouse IgG (1:500, abcam, ab150115). Nuclei were counterstained with DAPI. Imaged by confocal microscopy (TCS SP8, Leica). Uninfected cells served as negative controls.

### 2.10 Real‐Time Quantitative PCR

Total RNA was extracted using the RNeasy Mini Kit (QIAGEN, 74104). The NCBI's 69 FCV gene sequences were aligned with MEGA7.0, and primers and probes were designed using DNAMAN. After NCBI‐specific alignment, Beijing Qingke Synthetic synthesized the following primers: FCV‐F: GGRAARATTGTCAATGAHARBGT, FCV‐R: ACATCATATGCGGCTCTGA, and FCV‐P: FAM‐CCGCCAATCAACATGTGGTAA‐BHQ, with an amplification size of 129 bp. Absolute quantification was performed using 20 µL Hieff Unicon qPCR TaqManProbe Master Mix (Yeasen, 11205ES03). The FCV probe was commercially synthesized. The FCV copy number was determined using a standard curve.

### 2.11 Viral Infection Experiment and Clinical Scoring

The experimental cats were provided by the Institute of Animal Husbandry and Veterinary Medicine, Chinese Academy of Agricultural Sciences, and tested negative for FCV, parvovirus, herpesvirus, and infectious peritonitis virus. Nine 2‐month‐old domestic cats weighing 0.75–1.0 kg were randomly divided into three groups of three. Each group received 0.5 mL (0.2 mL per nasal passage, 0.05 mL per eye) of either 10^8^ TCID_50_/0.5 mL WT FCV‐BJ616 or rFCV‐BJ616 via intranasal and ocular routes, while the control group received an equivalent volume of saline. Clinical symptoms were recorded daily and scored on a 0–2 point scale for respiratory, oral, and ocular assessments. A double‐blind method was used to minimize bias (Table 2) (Luo et al. 2025). The infection cycle lasted 15 days, with daily symptom monitoring and temperature/weight measurements at days 0, 3, 6, 9, 12, and 15 post‐vaccination. Blood and serum samples were collected on days 0, 3, 9, and 15 for RT‐qPCR viral load analysis to confirm viremia. Eye and nasal swabs were also collected to detect viral shedding. On day 15, euthanasia was performed using 20% pentobarbital sodium (0.3 mL/kg) via intravenous injection according to World Animal Protection protocols. Lung, trachea, nasal mucosa, and throat samples were collected for histological and viral load analysis. If cats exhibited pain, immobility, or loss of appetite/drinking capacity during the experiment, humane euthanasia was administered.

**Table 2:** Standard for assessing clinical signs.

Clinical sign	Severity of symptoms	Score
Oral ulcer	Severity (large, ≥ 4 mm diameter)	2
	Slight (single or multiple, < 4 mm diameter)	1
	No	0
Eye and nose discharge	Severity	2
	Slight	1
	No	0
Body temperature	> 40°C or < 38°C	2
	38°C–38.5°C or 39.5°C–40°C	1
	38.5–39.5°C	0
Body weight	Reduction of 10% and above	2
	Within 10% reduction	1
	Steady increase	0
Mental state	Mental depression and loss of appetite	2
	Mental depression or loss of appetite	1
	Normal	0
Diarrhea	Severity	2
	Slight	1
	No	0
Other symptoms	Severity	2
	Slight	1
	No	0

### 2.12 Histopathological Observation

On day 15 post‐attack with WT FCV‐BJ616 or RFCV‐BJ616, lung, trachea, intestinal, liver, and kidney tissues were collected and fixed in 4% paraformaldehyde for 2 days. Following gradient alcohol dehydration and clearing, the tissues were embedded in paraffin and cut into 5 μm sections. These sections were then stained with hematoxylin and eosin (HE) for histological examination.

### 2.13 Proteomics Analysis

The proteomics methodology is based on prior studies (Zhu et al. 2020). In brief, proteins were extracted from frozen samples using lysis in 8 M urea, 1% SDS, and protease inhibitors, followed by multiple homogenization cycles and cryogenic sonication. Lysates were clarified by centrifugation, protein concentration was measured via BCA assay, and integrity checked by SDS‐PAGE. For digestion, 100 µg protein was adjusted to 100 mM TEAB, reduced, alkylated, centrifuged, resuspended, and digested overnight with trypsin. Peptides were dried, reconstituted, desalted, dried again, and quantified by NanoDrop. Equal peptide amounts were analyzed by UHPLC‐Orbitrap Astral MS using a uPAC column and specified solvents over 8 min, acquiring DIA data (m/z 150–2000). DIA files were processed in Spectronaut v19 with specific settings, FDR ≤ 1%, and MaxLFQ quantification. Statistical analyses used two‐sided *t*‐tests in R on the Majorbio Cloud platform. Differentially expressed proteins (fold change > 2 or < 0.5, *p* < 0.05) were GO/KEGG annotated and enriched(Ren et al. 2022).

### 2.14 Serum Cytokine ELISAs

The total serum IL‐8 and IFN‐β levels were measured using the ELISA kit (KIRbio) according to the protocol, with the average value calculated by the three‐well plate assay method.

### 2.15 Statistical Analysis

Bioinformatic analysis of proteomic data was performed with the Majorbio Cloud platform (https://cloud.majorbio.com). P‐values and Fold change (FC) for the proteins between the two groups were calculated using R package “*t*‐test.” The thresholds of fold change (1.2 or < 0.83) and *p*‐value < 0.05 were used to identify differentially expressed proteins (DEPs). Functional annotation of all identified proteins was performed using GO (http://geneontology.org/) and KEGG pathway (http://www.genome.jp/kegg/). DEPs were further used to for GO and KEGG enrichment analysis. Protein‐protein interaction analysis was performed using the String v11.5.

The SPSS software package (SPSS for Windows v13.0; SPSS Inc., Chicago, IL) was used to perform statistical analysis of the data obtained during the experiment. After normality testing, all data conformed to a normal distribution (Shapiro–Wilk test *p* > 0.05). Differences between the experimental and control groups were analyzed by Student's *t*‐test or one‐way ANOVA with Tukey's test using Prism 10 (GraphPad Software, San Diego, CA). Values are expressed in graph bars or a line graph as the mean ± SD of at least three independent biological replicates. *, *p* < 0.05; **, *p* < 0.01; and ***, *p* < 0.001 were considered statistically significant. ns, no significant difference.

## 3 Results

### 3.1 FCV Isolation and Identification

**Figure 1:**
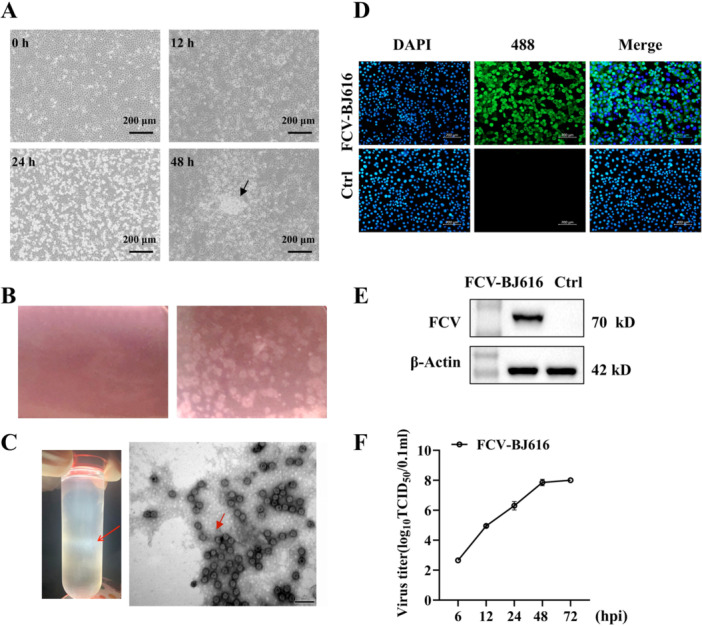
Isolation and Identification of FCV‐BJ616. (A) Cytopathic effects (CPE) observed in F81 cells infected at different time points (scale: 200 μm). (B) Results of third‐round plaque purification, with neomycin staining. (C) EM images of FCV‐BJ616 particles isolated from infected cultures, showing typical viral morphology. (D) IFA staining of FCV‐BJ616‐infected cells, showing green fluorescence from 488‐labeled viral particles and blue‐stained nuclei with DAPI. (E) Western blotting analysis of FCV‐BJ616 protein expression. (F) Validation of F81 cell susceptibility to FCV‐BJ616 infection, with viral titers measured by TCID_50_ at various time points.

To isolate FCV strains with replicative ability from clinical sources, this study inoculated suspected FCV samples into F81 cells. After cultivation, cytopathic effects (CPE), such as cell rounding, were observed (Figure 1A), followed by plaque purification to obtain cloned viruses (Figure 1B). TEM revealed that the virus was an envelope‐free spherical structure, approximately 35.6 nm in diameter, consistent with caliciviruses (Figure 1C). IFA and Western blotting confirmed the expression of the VP1 protein (Figure 1D,E). The virus titer was determined by TCID_50_ assay (Figure 1F).

### 3.2 FCV Whole Genome and Genetic Evolution Analysis

**Figure 2:**
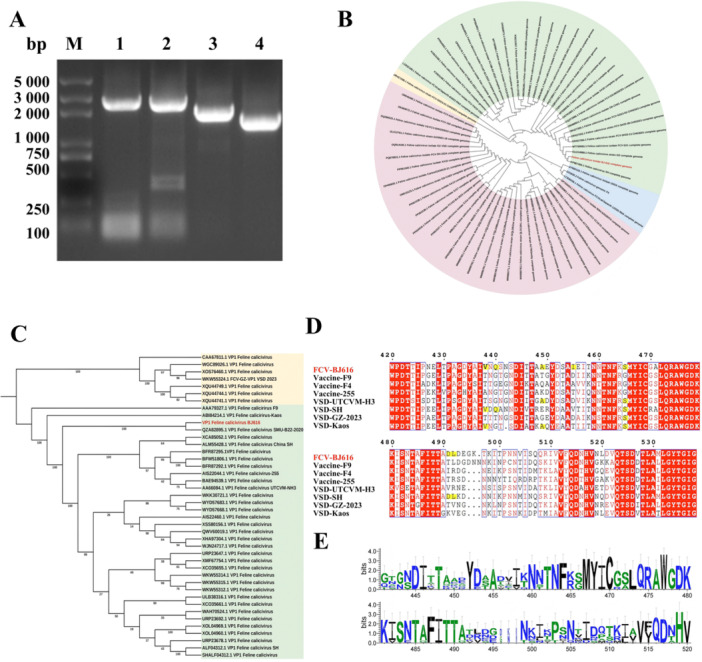
FCV‐BJ616 whole genome analysis and phylogenetic evolution analysis. (A) Whole genome PCR amplification of FCV‐BJ616. (B, C) Phylogenetic tree of nucleotide sequences from 69 FCV‐BJ616 strains, including both nucleotide and VP1 sequences. The tree was constructed using MEGA7.0 with the neighbor joining method and 1000 bootstrap replicates. The strain FCV‐BJ616 is highlighted in red. (D) Alignment and conservation analysis of key amino acid sites in the E region of FCV‐BJ616. In Figure D, the red background highlights highly conserved amino acids, while the yellow background indicates those matching the pathogenic sites of FCV‐VSD VP1. (E) Sequence identifiers of conserved amino acids across different domains (Note: Position y indicates the conservation level of amino acids at that position).

To investigate the molecular basis underlying the high pathogenicity of FCV‐BJ616, this study utilized three‐fragment overlapping PCR in combination with Illumina sequencing, resulting in the acquisition of a 7689 bp genome (Figure 2A). Phylogenetic analysis demonstrated that FCV‐BJ616 formed a distinct clade, separate from 63 globally circulating strains (Figure 2B), with its VP1 gene exhibiting the closest phylogenetic relationship to the highly virulent strain FCV SMU‐B22‐2020 (Figure 2C). Key Findings: Of the seven classical pathogenic sites within the VP1 high‐variability region, FCV‐BJ616 exhibited six VSD virulence‐associated mutations (438 V/440Q/448 A/455I/465S/492 L), while 430 T/443S/471 A/491D remained conserved relative to known virulent strains (Figure 2D). Despite high viral diversity, the E‐region showed strong conservation of the motifs (445‐450 N‐D‐I) (Figure 2E), further confirming FCV‐BJ616 as a novel variant with both VSD virulence potential and stable conserved domains. These findings led to the development of monoclonal antibodies targeting the VP1 high‐variability region, which demonstrated efficient neutralization and inhibition of viral replication (data not shown).

### 3.3 Construction of Infectious Clone of the FCV‐BJ616 Strain and Virus Rescue

**Figure 3:**
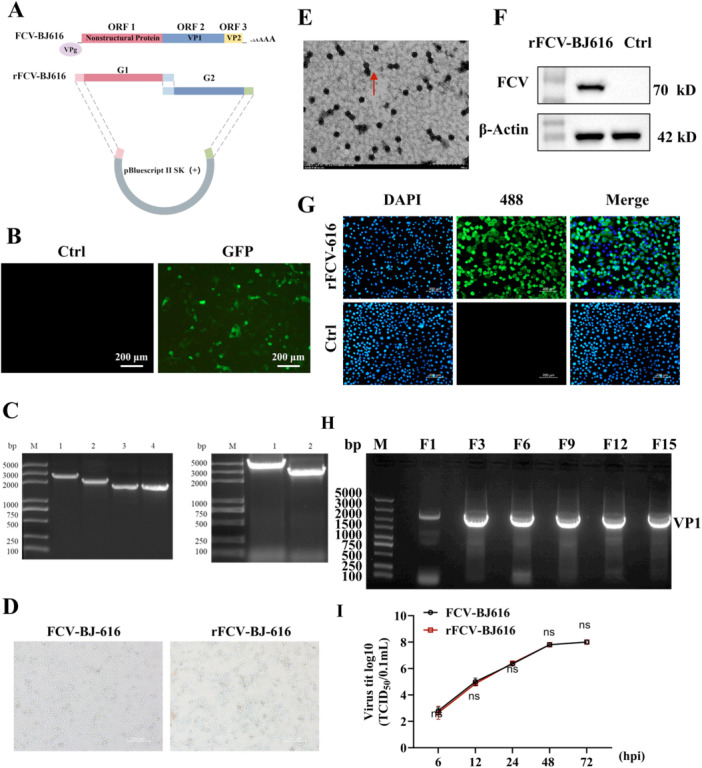
Construction of infectious clone FCV‐BJ616 and rescue of recombinant FCV‐BJ616 (rFCV‐BJ616). (A) Schematic diagram of constructing infectious clone FCV‐BJ‐616. (B) Transfection results of eGFP recombinant vector. (C) Full‐length RT‐PCR results for FCV‐BJ‐616. M: DL‐5000 DNA molecular weight standard. Left: g1 (2.5 kb), g2 (2 kb), g3 (1.6 kb), g4 (1.5 kb). Right: In‐Fusion PCR results for FCV‐BJ616. M: DL‐5000 DNA molecular weight standard, G1, G2. (D) Pathogenesis observation of rFCV‐BJ616. (E) TEM observation of viral particles of rFCV‐BJ616. (F, G) Western blotting and IFA detection of specificity of rFCV‐BJ616. (H) RT‐PCR detection of genetic stability of recombinant virus. F1‐F15 indicate passages to the 15th generation. (I) TCID_50_ test comparing the virulence of FCV‐BJ616 and rFCV‐BJ616. ns: no significant difference.

To establish a reverse genetics platform for FCV‐BJ616 and assess the properties of the recombinant virus, this study successfully constructed infectious clones and rescued the virus. Using reverse genetics techniques, we generated infectious clones (Figure 3A) and confirmed the expression potential of the modified vectors (Figure 3B). The amplification of g1‐g4 fragments (Figure 3C) via fusion PCR produced large G1 and G2 fragments, which were efficiently cloned into the recombinant plasmid pBluescript II SK (+) ‐G1G2 (Figure 3D). Transfection of F81 cells resulted in cytotoxic effects (Figure 3E). Following passage, the virus was successfully rescued, and electron microscopy confirmed the characteristic cup‐shaped morphology. Western blotting identified the target proteins (Figure 3F,G), while RT‐PCR confirmed stable expression of the VP1 gene (Figure 3H), with titers comparable to those of the parental virus (Figure 3I). These findings demonstrate the genetic stability and robust biological properties of the recombinant virus.

### 3.4 Clinical Symptoms and Viral Load in Cats Infected With FCV‐BJ616 or rFCV‐BJ616

**Figure 4:**
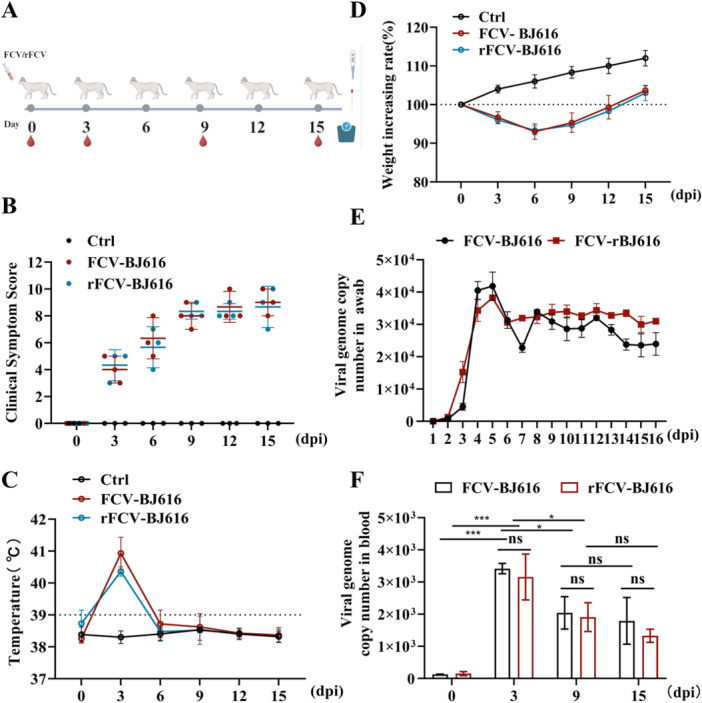
Evaluation of clinical symptoms and viral load after infection. (A) Experimental design schematic. Six time points during FCV‐BJ616/rFCV‐BJ616 infection, along with blood sampling procedures at each time point. (B) Clinical symptom scoring. Clinical symptom scores of cats in each group at different time points (days, dpi) after infection. (C) Body temperature changes. Temperature variations in cats across groups at different time points post‐infection. (D) Body weight change rate. Weight change rates in cats across groups at different time points post‐infection. (E) Viral genome copy counts in nasopharyngeal swab samples at different time points, expressed as the average number of copies per milliliter. (F) Viral genome copy count in blood. Viral genome copy counts in blood of cats in each group at days 3, 6, and 9 post‐infection (dpi). Data are represented as mean ± SD of three independent experiments. Student's *t*‐test or One‐way ANOVA test determined statistical. *p < 0.01; ***p < 0.001; significance: ns, not significant.

To assess whether rFCV‐BJ616 retains the pathogenicity and transmission dynamics of FCV‐BJ616, we monitored the cats for 15 days following infection with both strains. Oral/nasal swabs and blood samples were collected to analyze viral load (Figure 4A). The results revealed that all groups exhibited synchronized clinical deterioration (Figure 4B), sudden fever spikes (Figure 4C), and weight loss (Figure 4D) on day 3, with no significant differences between the groups. Viral dynamics were consistent across the groups: oral/nasal shedding peaked on day 3 before stabilizing (Figure 4E), while viremia peaked on day 3 and declined by day 9 (Figure 4F). In conclusion, the successful rescue of rFCV‐BJ616 and its demonstrated ability to cause disease confirm its pathogenic potential and provide a critical tool for future studies.

### 3.5 Systemic Multi‐Organ Pathology Assessment in Feline Infected With FCV‐BJ616 and rFCV‐Bj616

**Figure 5:**
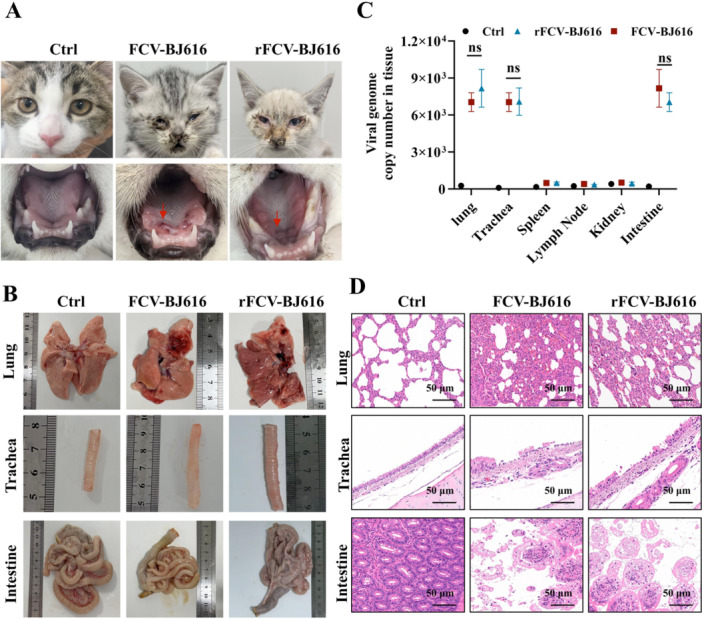
Pathological findings in cats infected with FCV‐BJ616 and rFCV‐BJ616. (A) Clinical manifestations observed in infected cats. (B) Pathological features of infected cats. (C) Viral load across different tissues. (D) Lung and lymph node histopathological sections from the control group.

To assess the pathogenicity of FCV‐BJ616 and its recombinant variant rFCV‐BJ616, cats were monitored for clinical signs and tissue lesions post‐infection. The results showed high fever, anorexia, lethargy and diarrhea from days 2 to 4, followed by conjunctivitis and severe oral ulcers on day 5 (Figure 5A). Macroscopic examination showed significant pulmonary congestion, tracheal epithelial damage with ciliary loss, intestinal inflammation, and lesions in other organs (Figure 5B and Figure S1A). Viral load analysis indicated higher viral loads in the lungs, trachea, and intestines, with no significant difference between the FCV‐BJ616 and rFCV‐BJ616 infection groups (Figure 5C). HE staining revealed severe microscopic lesions: thickened alveolar walls with interstitial edema and inflammatory cell infiltration in lung tissue; tracheal epithelial damage with ciliary loss; severe intestinal villus damage, necrosis with hemorrhage and inflammation; splenic lymph node swelling; and blurred glomeruli with tubular epithelial degeneration and necrosis accompanied by inflammatory cell infiltration in the kidneys (Figure 5D and Figure S1B). These findings suggest that FCV‐BJ616 and rFCV‐BJ616 infections induce widespread inflammation, structural damage, and cellular injury/necrosis across multiple organs (lung, trachea, spleen, lymph nodes, kidneys, intestines) in cats, providing critical pathological evidence for understanding pathogenic mechanisms and guiding therapeutic research.

### 3.6 FCV‐BJ616 and rFCV‐BJ616 Exhibit Similar Proteomic Differential Regulatory Effects

**Figure 6:**
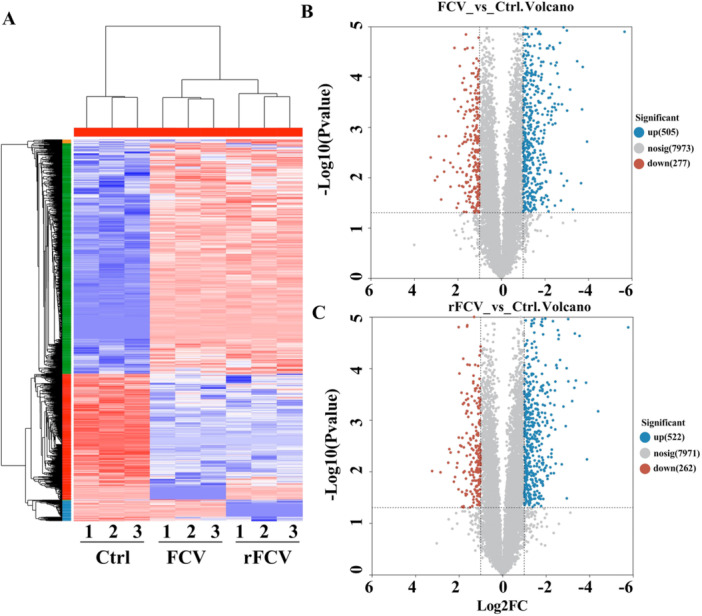
Heatmap and volcano plot of differentially expressed proteins in F81 cells infected with FCV‐BJ616 or rFCV‐BJ616. (A) The heatmap illustrates differences in gene expression among three groups: Ctrl, FCV (FCV‐BJ616), and rFCV (rFCV‐BJ616). Color intensity reflects gene expression levels, with red indicating up‐regulation and blue indicating downregulation. (B, C) Volcano plots comparing the FCV and rFCV groups relative to Ctrl. The X‐axis shows log2 fold change (Log2FC), and the Y‐axis displays ‐log10(*p*‐value). In both volcano plots, blue dots represent significantly upregulated genes (up), red dots represent significantly downregulated genes (down), and gray dots represent genes with no significant change (nosig). Figure B highlights differences between FCV and Ctrl groups, while Figure C demonstrates variations between rFCV and Ctrl groups.

To compare the overall regulatory effects of FCV‐BJ616 and rFCV‐BJ616 on the F81 cell proteome and evaluate the similarities and differences in the quantity and directionality of DEPs induced by the two viruses, this study conducted a systematic analysis using quantitative proteomics data. Heatmap analysis revealed a significant separation of samples from the Ctrl, FCV‐BJ616 and rFCV‐BJ616 groups according to the treatment conditions, with stable clustering patterns observed for both proteins and samples. This confirmed the systematic effects of treatment conditions on protein abundance profiles (Figure 6A). After applying a unified differential threshold (adjusted for multiple testing), 782 DEPs were identified in the FCV‐BJ616 group compared to the control group (505 upregulated, 277 downregulated), and 784 DEPs were identified in the rFCV‐BJ616 group (522 upregulated, 262 downregulated). The volcano plot analysis confirmed these statistical results (Figure 6B,C). Overall, the protein abundance changes induced by both treatments showed high similarity in scale and directionality, with the total number of DEPs differing by only two. Both groups predominantly showed significant upregulation, indicating comparable overall regulatory intensity of FCV‐BJ616 and rFCV‐BJ616 on the proteome. This finding lays the foundation for subsequent pathway enrichment analysis and the functional interpretation of common and specific DEPs.

### 3.7 Enrichment Analysis Results for GO and KEGG

**Figure 7:**
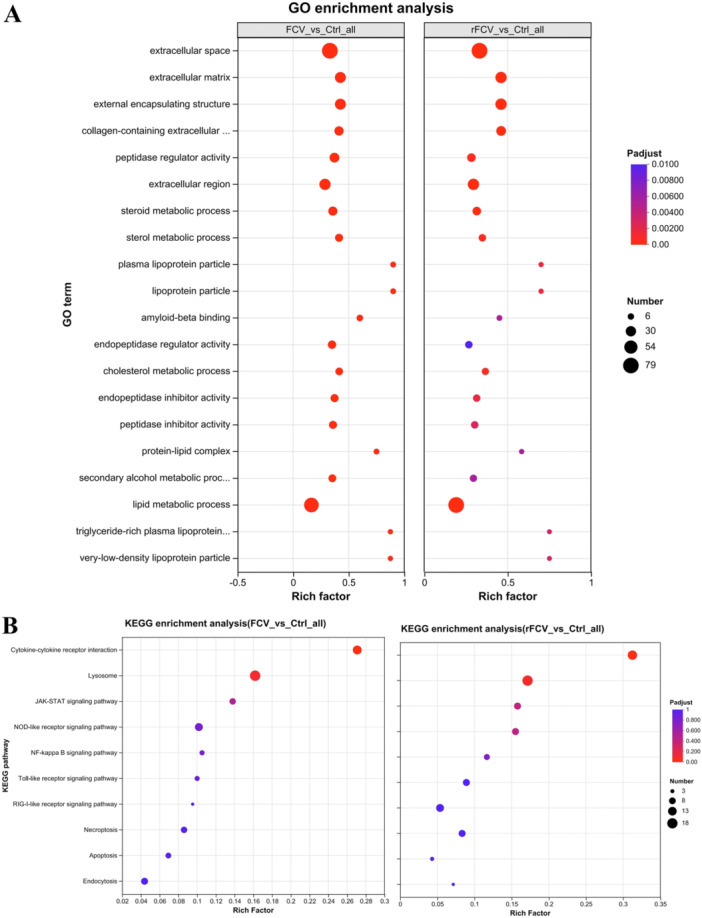
GO and KEGG enrichment analysis of differentially expressed proteins. (A) GO enrichment analysis. The bubble color indicates the adjusted *p*‐value (Padjust), with colors transitioning from blue to red indicating decreasing *p*‐values; the bubble size reflects the number of genes (Number). (B) KEGG enrichment analysis. The bubble color indicates the adjusted *p*‐value (Padjust), with colors transitioning from blue to red indicating decreasing *p*‐values; the bubble size reflects the number of genes (Number).

To elucidate the regulatory mechanisms of FCV‐BJ616 and rFCV‐BJ616 on host cells, this study performed GO and KEGG enrichment analyses. The results revealed striking similarities in the regulation of core signaling pathways by both viral strains. GO analysis identified significant enrichment of FCV‐BJ616 and rFCV‐BJ616 in pathways associated with extracellular matrix remodeling and sterol metabolism reprogramming (corrected *p*‐value < 0.01), suggesting that FCV strains can disrupt tissue structure and interfere with lipid homeostasis (Figure 7A). KEGG analysis further confirmed significant activation of cytokine‐cytokine receptor interactions, lysosomal function, and JAK‐STAT signaling pathways in both viral strains (corrected *p*‐value < 0.005), with consistent enrichment patterns that were significantly different from the control groups. This suggests that the virus may hijack lysosomes through conserved mechanisms (mediating cell invasion), suppress JAK‐STAT pathways (facilitating immune evasion), and trigger cytokine storms (driving inflammatory responses) (Figure 7B). The conserved regulatory patterns of FCV‐BJ616 and rFCV‐BJ616 on these core pathways reflect the genetic stability of the pathogenic mechanisms in this viral genus. Therefore, the rFCV‐BJ616 strain can serve as an effective reverse genetics platform for in‐depth analysis of these conserved mechanisms, providing a theoretical foundation for the development of broad‐spectrum antiviral drugs and vaccines targeting these pathways.

### 3.8 IL‐8 and IFN‐β Are Key Mediators in Cytokine Storms

**Figure 8:**
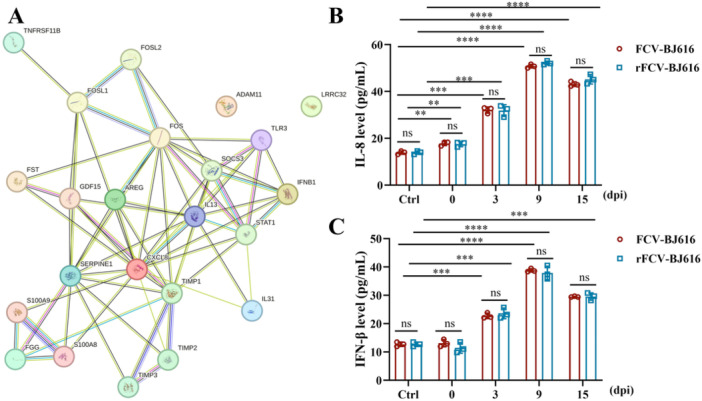
The dynamic expression of IL‐8 and IFN‐β in cat serum. (A) PPI network analysis diagram of inflammatory‐related factors. (B, C) ELISA analysis of IL‐8 and IFN‐β expression at different time points following infection with FCV‐BJ616 and rFCV‐BJ616. Data are represented as mean ± SD from three independent experiments. Statistical significance was determined by Student's *t*‐test: ***p < 0.001; **p < 0.01; ns, not significant.

To evaluate the inflammatory responses caused by FCV‐BJ616 and rFCV‐BJ616 infections, we analyzed and integrated inflammation‐related factors identified through omics in cells infected with FCV‐BJ616 and rFCV‐BJ616, including inflammatory factors such as IL‐8, IFN‐β, and TGF‐β (Supporting Information Table S1). The PPI network analysis revealed their mutual regulatory interactions (Figure 8A). Serum samples were collected from infected cats on days 0, 3, 9, and 15 post‐infection, and the levels of key pro‐inflammatory cytokines IL‐8 and IFN‐β were measured using ELISA kits. The results revealed significantly higher serum concentrations of IL‐8 and IFN‐β in the FCV‐BJ616/rFCV‐BJ616 infected group compared to healthy controls (*p* < 0.05) (Figure 8B,C). These data suggest that IL‐8 might mediate systemic inflammatory responses, thereby contributing to the development and progression of FCV‐associated oral lesions (e.g., oral ulcers) and skin damage. These findings are consistent with the significantly activated “cytokine‐receptor interaction” pathway identified in transcriptome enrichment analysis, further reinforcing the central pathogenic role of cytokine storms in FCV infection.

## 4 Discussion

FCV is the primary pathogen responsible for feline upper respiratory disease (URTD), vasculitis, and systemic disease (VSD) (Bordicchia et al. 2021). The absence of a proofreading function in its RNA polymerase results in high genetic variability, which presents challenges for vaccine development. Despite the availability of several FCV vaccines, such as F9, 21,255,431, and G1, the virus's high mutation rate and frequent outbreaks in China have raised concerns regarding the effectiveness of these vaccines (Cao et al. 2023; Gao et al. 2023; Mao et al. 2022; S. J. Zhang et al. 2025). This study isolated and identified a highly pathogenic VSD strain, FCV‐BJ616. Sequence analysis revealed that its VP1 protein carries six VSD‐specific mutations (438 V/440Q/448 A/455I/465S/492 L) and highly conserved residues (430 T/443S/471 A/491D), confirming VP1 as a key virulence factor. The clinical symptoms caused by this gene are highly consistent with those of high virulence strains, which explains its severe clinical manifestations (oral ulcers, systemic inflammation) and high viral load in tissues (Luo et al. 2025; Park et al. 2024). Notably, the conserved motif (445‐450 N‐D‐I) in the VP1 high‐variability region may serve as a structural anchor for immune evasion or provide a potential target for broad‐spectrum antiviral drug design.

Using reverse genetics, the study successfully generated the rescue virus rFCV‐BJ616. Similarly, researchers using a methodology similar to that of this study successfully rescued rFCV‐△VP2, which conferred robust immune protection against FCV(Heng et al. 2025). However, the rescued virus strain rFCV‐BJ616 exhibited distinct genotypes and stable phenotypes, effectively overcoming the virulence decline observed in wild‐type isolates during successive passages. It maintains over 99.8% genomic fidelity even after multiple passages while consistently inducing classic VSD symptoms in vivo: persistent fever, multi‐organ pathology, and cytokine storm. Therefore, the recombinant virus strain rFCV‐BJ616 developed in this study is a valuable tool for investigating the pathogenic mechanisms of FCV, and it holds potential for use in targeted mutations of VP1 to develop attenuated vaccines.

Traditional classification divides FCV into respiratory (R‐FCV) and enteric (E‐FCV) subtypes based on isolation sites, with the latter exhibiting bile acid resistance(Di Martino et al. 2020; Guo et al. 2022). Our study revealed that FCV‐BJ616 maintained persistent viral RNA in oral/anal swabs and blood throughout the infection period. Tissue viral load analysis demonstrated high viral replication in both respiratory (lung, trachea) and enteric tissues. Although FCV is well‐known as a respiratory pathogen, its intestinal pathogenicity remains underexplored, with few models correlating FCV with gastrointestinal symptoms (S. J. Kim et al. 2021; Z. Wang et al. 2021). This study notably demonstrated that both FCV‐BJ616 and rFCV‐BJ616 induced severe diarrhea, mild rectal prolapse accompanied by petechiae, and intestinal wall thickening. H&E staining revealed extensive infiltration of macrophages in the intestinal tissues, suggesting direct viral damage. These findings are also consistent with previously reported pathogenic phenotypes of intestinal isolates causing enteritis (Luo et al. 2025). Therefore, further elucidation of the organizational tendencies and virulence evolution of FCV is crucial.

Currently, FCV research is primarily focused on epidemiology (S. Kim et al. 2024; Komina et al. 2022; Sun et al. 2017), while investigation into its pathogenic mechanisms remains comparatively limited (Mao et al. 2023; Peñaflor‐Téllez et al. 2024; Pérez‐Ibáñez et al. 2024; Tian et al. 2020; Wu et al. 2021). This study employs proteomics, a technology increasingly vital for studying viral pathogenesis (Chen et al. 2025; Harsha et al. 2022; Zhou et al. 2011). combined with KEGG pathway enrichment analysis to systematically elucidate the core pathogenic mechanisms of FCV infection. Our findings demonstrate that FCV proteins disrupt key signaling pathways, including RIG‐I, JAK‐STAT, and NF‐κB, thereby inhibiting interferon production and signal transduction (Liu et al. 2025; Peralta et al. 2023; Tian et al. 2020). Proteomic data indicate that FCV‐BJ616 infection engages TLR3 signaling and is associated with increased IFN‐β, while STAT1 abundance or activity appears reduced. Together, these findings are compatible with type I IFN induction alongside attenuated downstream JAK‐STAT signaling, which may limit the expression of interferon‐stimulated genes. This pattern suggests heightened inflammation with comparatively weak antiviral signaling, potentially allowing ongoing viral replication. Inflammatory mediators such as IL‐8 and S100A8/A9 were also upregulated, consistent with tissue‐damaging inflammation and multiorgan involvement observed in vivo. These interpretations are provisional and based on proteomic and ELISA data; comprehensive immune profiling will be required to confirm mechanisms and establish causality.

Although we successfully established a reverse‐genetics platform for FCV‐BJ616 and elucidated key aspects of its pathogenesis, several limitations warrant further investigation. First, the genetic stability of the cloned virus remains insufficiently characterized, as evaluations of rFCV‐BJ616 were largely confined to early passages. Comprehensive long‐term serial passaging, deep sequencing, and rigorous phenotypic profiling are needed to monitor evolutionary dynamics and assess the risk of reversion to virulence, thereby supporting its safety assessment as a vaccine candidate. Second, the in vivo study design was constrained by small sample sizes (*n* = 3 per group) and a single age cohort. Future studies should include larger, more diverse cohorts spanning multiple age ranges and physiological states and incorporate in vivo immunological assessments. Finally, mechanistic validation of key mediators (e.g., IL‐8 and IFN‐β) remains preliminary, and the causality and hierarchical regulation underlying events such as STAT1 downregulation are not yet defined. Targeted loss‐ and gain‐of‐function experiments, including the use of specific inhibitors, are needed to establish causality and delineate their contributions to pathogenicity.

In conclusion, this study elucidates the molecular mechanisms underlying FCV virulence and establishes a robust reverse genetics platform for the virus. The successful rescue of rFCV‐BJ616 not only enables precise genetic manipulation and functional dissection of viral genes but also provides a powerful tool for vaccine strain design and antiviral research. Integrating multi‐omics and pathological analyses, this work advances our understanding of FCV pathogenesis and offers theoretical and experimental foundations for developing next‐generation, broad‐spectrum FCV vaccines and therapeutic interventions.

## Author Contributions

**Chunmei Xu:** writing – review and editing, writing – original draft, methodology, formal analysis, data curation. **Jingjie Zhao:** methodology, formal analysis, investigation. **Hao Liu:** investigation. **Haotian Gu:** investigation. **Xinming Tang:** investigation. **Lin Liang:** investigation. **Jiabo Ding:** supervision, **Shaohua Hou:** supervision, **Xiaomin Zhao:** writing – review and editing. **Ruiying Liang:** writing – review and editing, project administration, funding acquisition.

## Ethics Statement

All animal experiments were conducted under protocols by the Science Research Department (in charge of animal welfare issues) of the Institute of Animal Science, Chinese Academy of Agricultural Sciences (No IAS2025‐138). Research was conducted in compliance with the principles stated in the Guide for the Care and Use of Laboratory Animals, National Research Council, 1996.

## Conflicts of Interest

The authors declare no conflicts of interest.

## Supporting information


**Figure S1:** Pathological anatomical changes induced by FCV‐BJ616 and rFCV‐BJ616 infection (A) and HE staining analysis (B).

**Table S1:** Host inflammatory factors co‐regulated by FCV‐BJ616 and rFCV‐BJ616 infections.

## Data Availability

The mass spectrometry proteomics data have been deposited to the ProteomeXchange Consortium via the PRIDE (Perez‐Riverol et al. 2025) partner repository with the dataset identifier PXD070016.
