# Supplementary figures and images for: Construction of Reverse Genetics System for Feline Calicivirus FCV‐BJ616 and Proteomic Analysis
Source: Microbiologyopen. 2026 Jan 28;15(1):e70226. doi: 10.1002/mbo3.70226 (PMC12849204; doi:10.1002/mbo3.70226)

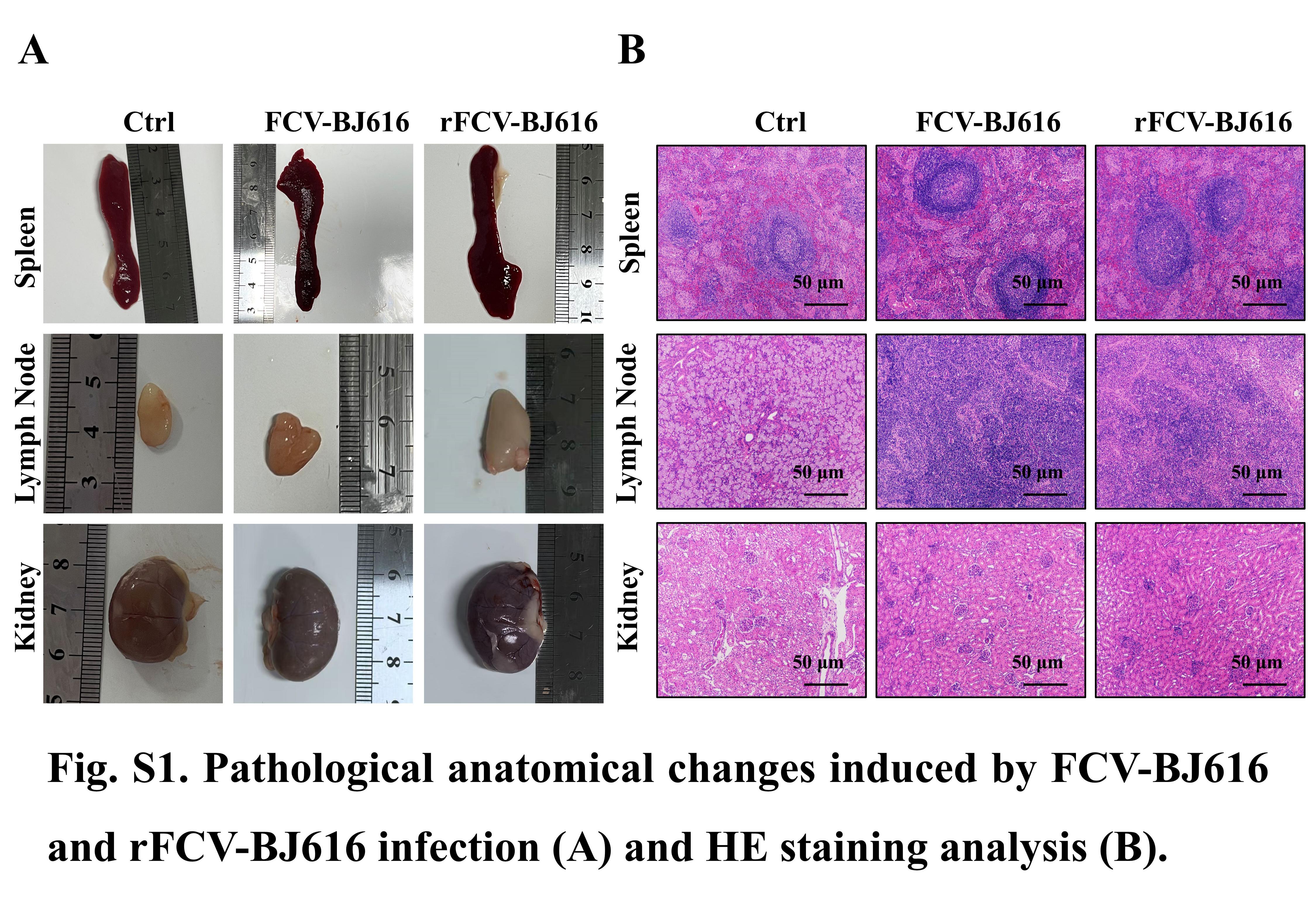

Supplement: Supplementary file 1 — Figure S1: Pathological anatomical changes induced by FCV‐BJ616 and rFCV‐BJ616 infection (A) and HE staining analysis (B). [file MBO3-15-e70226-s002.JPG]
