# Supplementary material for: Construction of Reverse Genetics System for Feline Calicivirus FCV‐BJ616 and Proteomic Analysis
Source: Microbiologyopen. 2026 Jan 28;15(1):e70226. doi: 10.1002/mbo3.70226 (PMC12849204; doi:10.1002/mbo3.70226)
Supplement: Supplementary file 2 — Table S1: Host inflammatory factors co‐regulated by FCV‐BJ616 and rFCV‐BJ616 infections. [file MBO3-15-e70226-s001.docx]

**Table S1 Host inflammatory factors co-regulated by FCV-BJ616 and rFCV-BJ616 infections**

| **Molecular name** | **classification** | **Functional description** |  |
| --- | --- | --- | --- |
| IL-8 | Chemokine | Major neutrophil chemoattractant, promotes acute inflammation |  |
| MDC | Chemokine | Recruits regulatory T cells (Tregs) to inflammation sites |  |
| IFN-β | Type I Interferon | Key antiviral cytokine, activates immune cells via JAK-STAT pathway |  |
| TGFβ1 | Pleiotropic cytokine | Dual role: early pro-inflammatory, late anti-inflammatory/tissue fibrosis |  |
| TGFβ2 | Pleiotropic cytokine | TGF-β family member with similar functions to TGFB1 |  |
| Amphiregulin | EGF-family growth factor | Tissue repair mediator, epithelial regeneration during inflammation |  |
| FGG | Acute-phase protein | Coagulation factor with pro-inflammatory cleavage products |  |
| PAI-1 | Serine protease inhibito | Regulates fibrinolysis, promotes fibrosis in chronic inflammation |  |
| IL-31 | Cytokine Receptor | Mediates IL-31 signaling (pruritus/allergic inflammation) |  |
| IL-13 | Decoy Receptor | Binds IL-13 to regulate Th2 inflammation |  |
| TLR3 | Pattern Recognition Receptor | Detects viral RNA, activates interferon/NF-κB pathways |  |
| S100A8 | Alarmin | Calprotectin component (neutrophil-driven inflammation)ok |  |
| S100A9 | Alarmin | Calprotectin subunit (amplifies inflammatory response) |  |
| GDF15 | Stress Cytokine | Marker of disease cachexia and metabolic stress |  |
| FST | Activin Inhibitor | Regulates tissue repair and fibrosis resolution |  |
| FOS | Transcription Factor (AP-1) | Controls IL-6/TNF expression in early inflammation |  |
| FOSL1 | Transcription Factor (AP-1) | Activates inflammatory gene transcription |  |
| FOSL2 | Transcription Factor (AP-1) | Regulates MMPs/cytokine production in chronic inflammation |  |
| SOCS3 | Signaling Suppresso | Inhibits JAK-STAT pathway (negative feedback) |  |
| STAT1 | Transcription Factor | Core mediator of interferon signaling |  |
| TIMP1 | Metalloproteinase Inhibitor | Controls inflammatory mediator activity and ECM remodeling |  |
| TIMP2 | Metalloproteinase Inhibitor | Regulates tissue remodeling and fibrosis |  |
| TIMP3 | Metalloproteinase Inhibitor | Modulates vascular inflammation and stability |  |
| TNFRSF11B | TNF Receptor Superfamily | Osteoprotegerin (OPG) - regulates bone/vascular inflammation |  |
